# Extramedullary Hematopoiesis in Mismatch Repair Deficient Colon Cancer Patient on Adjuvant Chemotherapy

**DOI:** 10.7759/cureus.12899

**Published:** 2021-01-25

**Authors:** Edward K De Leo, Chintan P Shah, Joseph R Grajo, Xiuli Liu, Hiral Parekh

**Affiliations:** 1 Internal Medicine, University of Florida, Gainesville, USA; 2 Hematology and Oncology, University of Florida, Gainesville, USA; 3 Radiology, University of Florida, Gainesville, USA; 4 Pathology, University of Florida, Gainesville, USA; 5 Oncology, Cancer Specialist of North Florida, Jacksonville, USA

**Keywords:** mmr deficient, msi- high, extramedullary hematopoiesis, colon cancer

## Abstract

A 59-year-old male presented with a two-month history of abdominal pain and was found to have an obstructing cecal mass. Colonoscopy and biopsy confirmed invasive adenocarcinoma. Immunohistochemical analyses for mismatch repair (MMR) proteins revealed the loss of MLH1 as well as PMS2 in cancerous nuclei, which makes the tumor MMR deficient. Negative germline testing for MMR proteins ruled out the Lynch syndrome. After negative staging computerized tomography scan for distant metastases, he underwent ileocolectomy with ileotransverse colonic anastomosis. Final pathological analysis revealed poorly differentiated adenocarcinoma with signet ring features, negative margins, and 3/22 lymph nodes positive, classified as stage IIIB (T4aN1bM0). Adjuvant chemotherapy with modified FOLFOX (leucovorin calcium/folinic acid, fluorouracil, and oxaliplatin) was started without the use of any growth factor support. After cycle 9 of 12, he developed mild transaminitis, carcinoembryonic antigen elevation, and interval development of two heterogeneously enhancing hepatic lesions. Biopsy of both of these lesions revealed extramedullary hematopoiesis (EMH), with no evidence of metastatic disease. He completed adjuvant chemotherapy without complication, and these liver lesions have decreased in size during the follow-up period of almost two years thus far.

EMH is extremely rare in patients with colon cancer. Contributing factors include therapy-specific (growth factor support), bone marrow suppression secondary to chemotherapy and radiation therapy, and tumor-specific factors (cytokine and growth factors released by the tumor). To the best of our knowledge, this is the first case report of EMH in an MMR deficient colon cancer patient on adjuvant FOLFOX. MMR-deficient tumors show signs of a high degree of infiltration with CD8+ cytotoxic T lymphocytes as well as helper T cells, leading to increased production of cytokines, such as interferon-γ. This could be a potential etiology behind EMH in our patient who was MMR deficient. The role of the MMR-deficient state in the development of EMH should be explored further.

## Introduction

Extramedullary hematopoiesis (EMH) is defined as hematopoiesis occurring in areas outside of the bone marrow, most commonly the liver and spleen. This process is physiologic during fetal development but pathologic in adult life and is usually a compensatory mechanism secondary to bone marrow malfunction [1]. It is most commonly seen in hematological diseases, such as thalassemia or hemolytic anemias, and bone marrow disorders, classically myelofibrosis [1]. Rarely, EMH has been described to occur in the presence of solid tumors, with breast cancer being the most common [2]. The clinical presentation of EMH is wide-ranging and dependent on the location of EMH; most are found in asymptomatic patients on routine imaging, while others can have location-specific presentations such as abdominal pain, respiratory symptoms, or even cord compression [3]. Radiographically, EMH lesions can appear as solitary or multiple masses, and can easily be mistaken for metastatic lesions in patients undergoing treatment for a solid tumor malignancy, which can have treatment altering consequences. Here, we describe a case of EMH discovered in a patient undergoing adjuvant chemotherapy for adenocarcinoma of the colon after resection of the primary tumor.

## Case presentation

**Figure 1:**
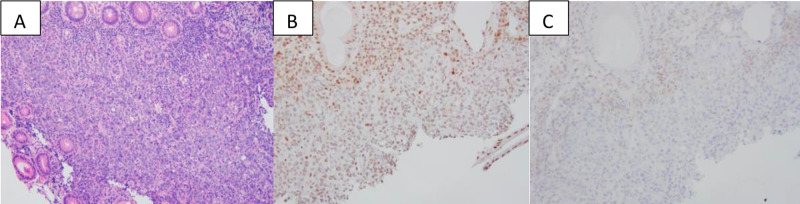
Immunohistochemical analyses of the biopsied colonic adenocarcinoma Biopsy of the cecal mass reveals sheet of cancerous cells (A, H&E stain). The cells show loss of nuclear immunoreactivity for MLH1 (B, immunostain) and PMS2 (C, immunostain) in the cancer.  The overall features support a diagnosis of colonic adenocarcinoma with mismatch repair deficient.

A 59-year-old Caucasian male presented with a two-month history of intermittent generalized abdominal pain and one week of diarrhea with bright red blood per rectum. His past medical, surgical, and family history was unremarkable. Contrast-enhanced computed tomography (CT) scan of the abdomen and pelvis showed an 8.2 x 7.6 x 9.0 cm cecal mass that involved terminal ileum, causing a small bowel obstruction, with multiple prominent lymph nodes noted in the right lower quadrant, without evidence of bowel perforation or distant metastasis. A colonoscopy revealed an obstructing cecal mass and biopsy confirmed invasive adenocarcinoma, positive for CK20, CDX2, and SATB2, but negative for CK7, compatible with colon primary. Immunohistochemical analyses of the biopsied colonic adenocarcinoma for mismatch repair (MMR) proteins revealed the loss of MLH1 and PMS2 in cancerous nuclei, making the tumor MMR deficient (Figure 1).

**Figure 2:**
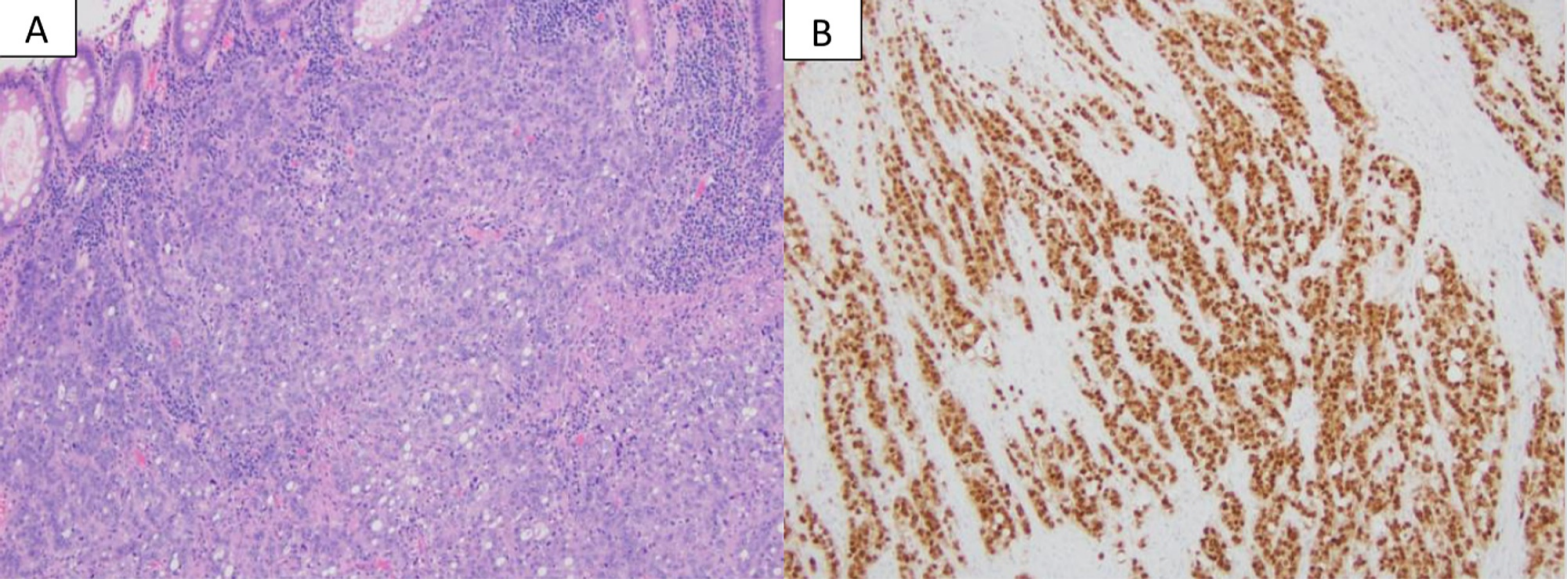
Pathological analysis of the surgical specimen Resection reveals a poorly differentiated adenocarcinoma with a solid and vague glandular growth pattern with many tumor infiltrating lymphocytes, features of mismatch repair deficient carcinoma (A, H&E stain). The tumor cells are strongly positive for CDX2 (B, immunostain), supporting a diagnosis of colonic adenocarcinoma.

Germline testing for these genes was negative, ruling out Lynch syndrome. His carcinoembryonic antigen (CEA) level was within the reference range at 1 ng/ml (0-3 ng/ml) and high sensitivity C-reactive protein was significantly elevated at 47.5 mg/l (0-5 mg/l). A laparoscopic ileocolectomy with ileotransverse colonic anastomosis was performed without any complications. Pathological analysis of the surgical specimen revealed poorly differentiated adenocarcinoma (Figure 2) with signet ring features, negative margins, and 3/22 lymph nodes positive. 

His pathological staging was classified as stage IIIB (T4aN1bM0) according to the 7th edition of the American Joint Committee on Cancer staging guidelines. His CEA level postoperatively was 2.6 ng/mL. After resection of the primary tumor, he was started on adjuvant chemotherapy with modified FOLFOX6 (leucovorin calcium/folinic acid, fluorouracil, and oxaliplatin) for a total of 12 cycles after multidisciplinary tumor board discussion.

**Figure 3:**
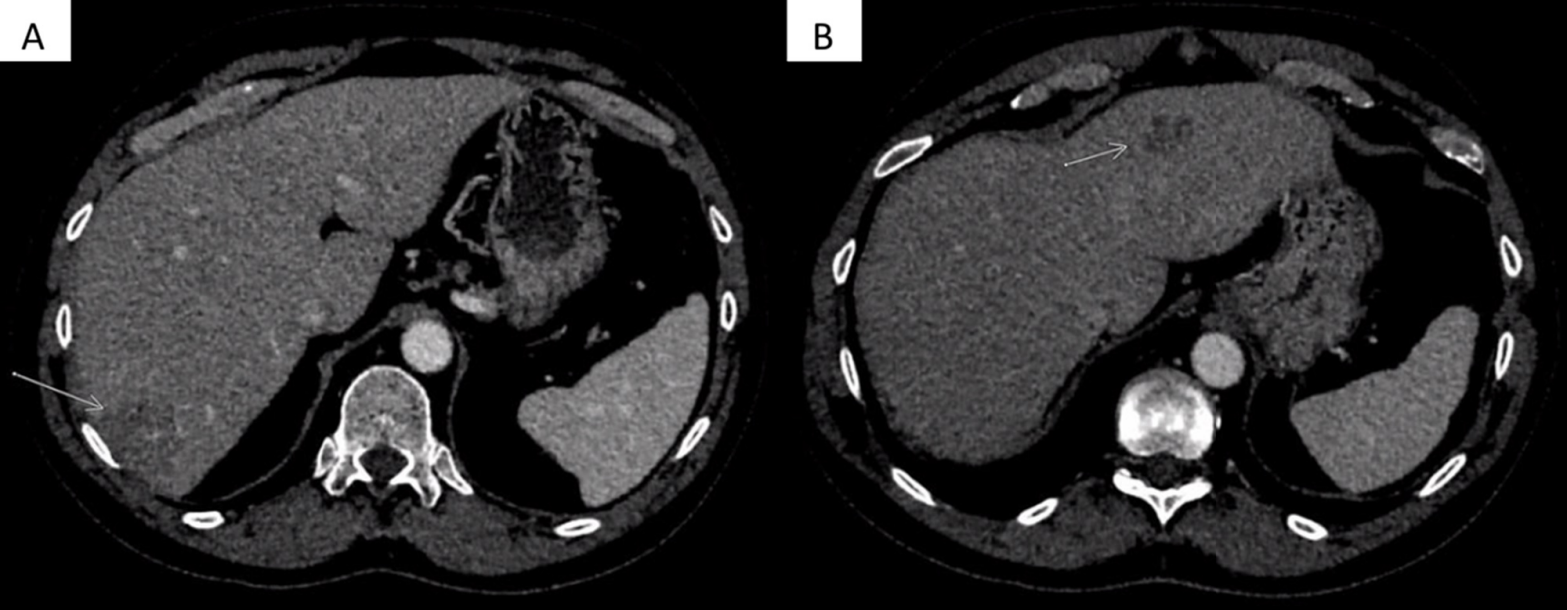
Interim CT images while on adjuvant chemotherapy Axial contrast-enhanced CT images through the liver demonstrate a discrete appearance of the low attenuating mass in the right hepatic lobe (arrow in A) seen on prior MRI. In addition, there is a new, smaller mass in the left hepatic lobe (arrow in B) that was not seen on prior imaging.

Over the initial five months of his adjuvant chemotherapy, he was noted to have a minimal but persistently rising CEA level from 2.6 ng/mL at the start of chemotherapy to 7.3 ng/mL after completing 9 of 12 planned cycles of chemotherapy. His complete blood count at this time was within the reference range. His aspartate aminotransferase (AST) was 46 IU/L and alanine aminotransferase (ALT) was 43 IU/L, mildly elevated since the start of chemotherapy. As a result of the rising CEA, a CT scan of the abdomen and pelvis with intravenous contrast was obtained, which revealed interval development of two heterogeneously enhancing masses in the right and left hepatic lobe, concerning metastatic disease (Figure 3A, 3B).

**Figure 4:**
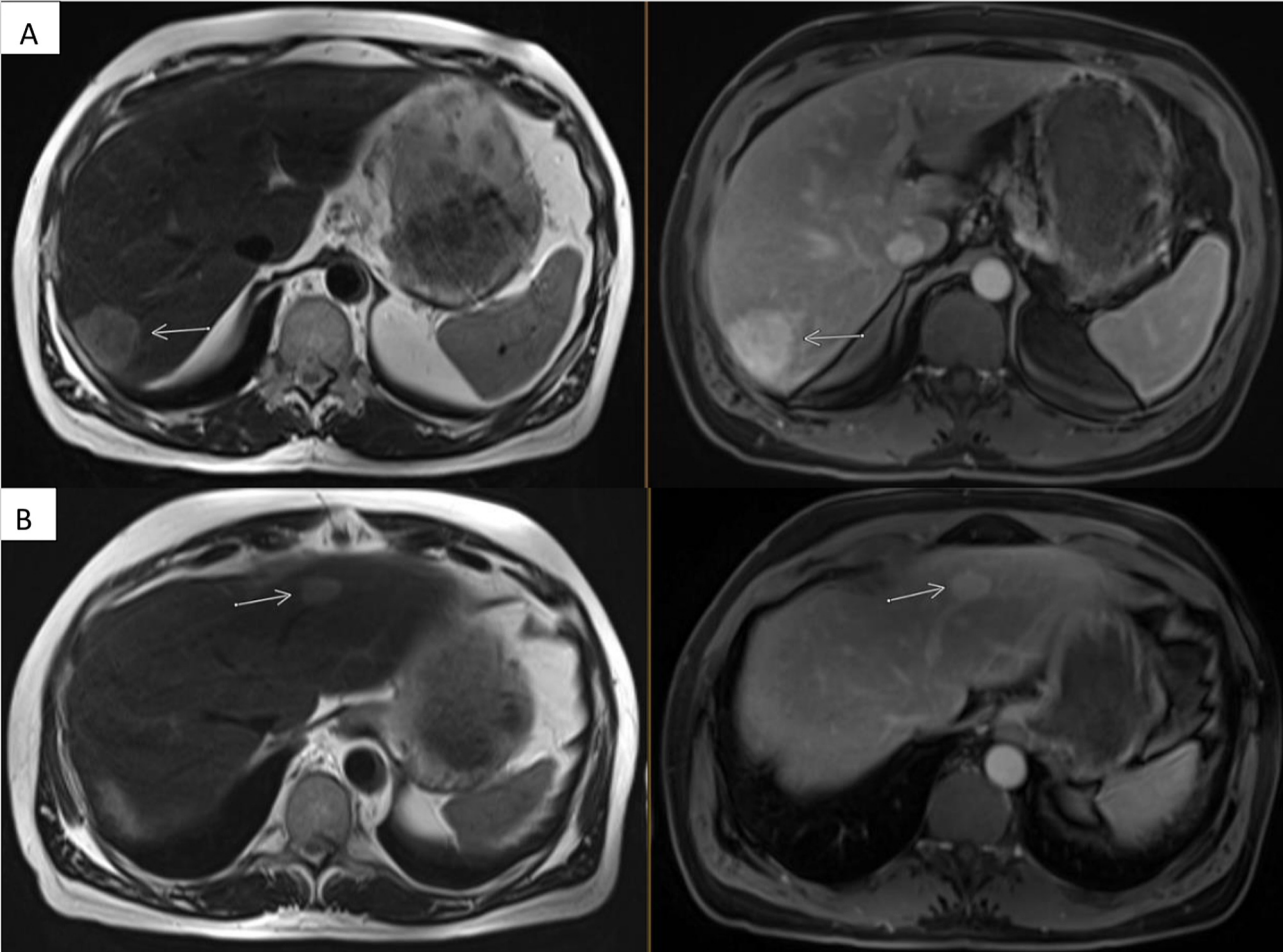
Interim MRI images while on adjuvant chemotherapy A: Axial T2 weighted image through the liver demonstrates increased T2 signal and conspicuity of the right hepatic lobe mass (arrow, left). Contrast-enhanced imaging was performed with an extracellular agent (gadobutrol) for this MRI. A three-minute post-contrast equilibrium phase image in the axial plane shows delayed enhancement of the mass (arrow, right). B: Axial T2 weighted image and three-minute post-contrast equilibrium phase showing similar T2 hyperintensity and delayed enhancement in the left hepatic lobe mass (arrows).

The MRI (Figure 4A, 4B) showed two T2 hyperintense, T1 hypointense hepatic lesions corresponding to the size and location as that in the CT scan.

**Figure 5:**
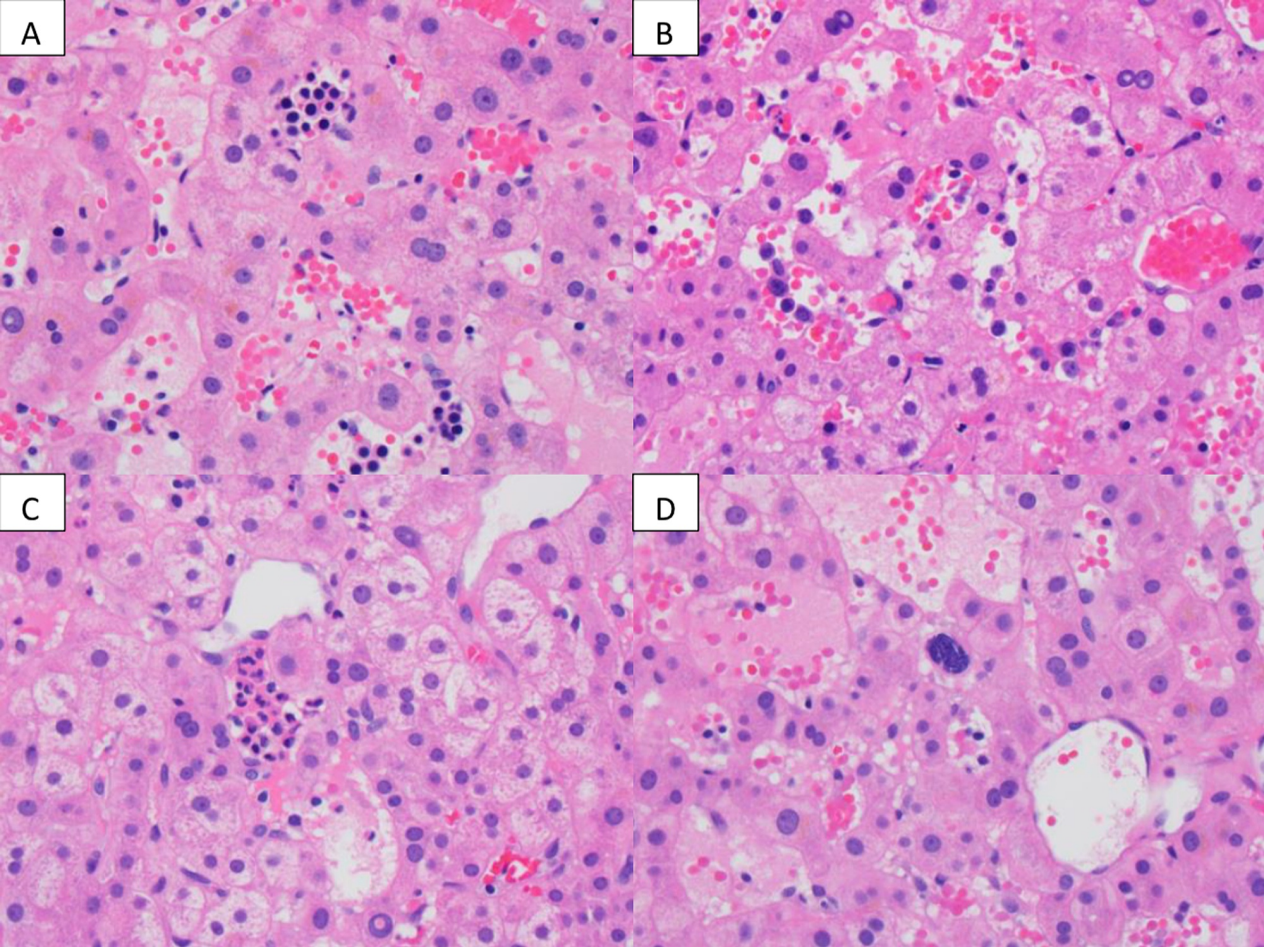
Liver biopsy from the right lobe mass and the left lobe mass Liver biopsy from the right lobe mass and the left lobe mass shows similar features including sinusoidal dilatation, congestion, and presence of erythroblasts (A, clusters of cells containing dark nucleus in the sinusoids ), maturing myeloid precursors (B, individual large cells in the sinusoids), and granulocytes (C, clusters in the center). Rare megakaryocytes (D, center of the picture) are also noted. Some of the sinusoids lack endothelial lining (D). These features support a diagnosis of extramedullary hematopoiesis with peliosis.

Both lesions exhibited delayed enhancement on MRI, which is not consistent with metastatic colon cancer. CT guided percutaneous needle core biopsy of both liver lesions revealed extramedullary hematopoiesis with peliosis. More specifically, the hepatic parenchyma demonstrated marked sinusoidal dilation, congestion, and hemorrhage with associated clusters of immature erythroid and myeloid precursors with rare megakaryocytes in the dilated sinusoids (Figure 5). 

**Figure 6:**
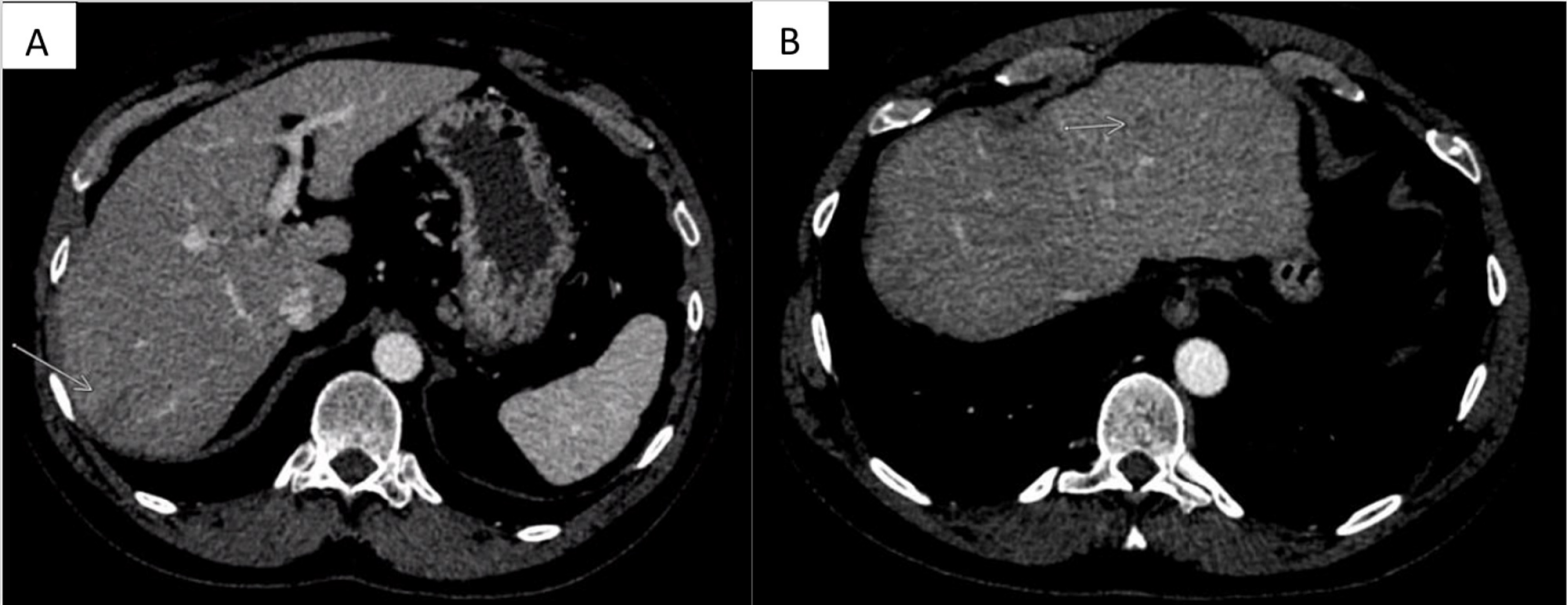
One-year post-treatment follow up CT images Axial post-contrast CT through the liver shows that the right hepatic lobe mass has decreased in size and conspicuity (arrow in A). The left hepatic lobe mass has also decreased in size and is now barely visible (arrow in B).

Some of the sinusoids were devoid of endothelial lining (Figure 5D). There was no evidence of malignancy or metastatic disease. The patient completed his final three cycles of chemotherapy for a total of six months of adjuvant systemic chemotherapy. Thus far, he has been followed for almost two years post-treatment. He remains asymptomatic, and the liver lesions from biopsy-proven extramedullary hematopoiesis have decreased in size (Figure 6A, 6B). 

He has no new findings on imaging since treatment ended. His labs at last visit included white blood cells 5.9 (4-10 thou/cu mm), hemoglobin 15.7 (13-16.5 g/dL), platelets 281 (150-450 thou/cc mm, AST (aspartate aminotransferase) 25 (0-37 IU/L), and ALT (alanine aminotransferase) 29 (0-50 IU/L). His CEA peaked at 7.3 ng/mL after cycle 9 of modified FOLFOX6 and then slowly trended down and has remained below the reference range (< 3 ng/mL).

## Discussion

To the best of our knowledge, this is the first case report of EMH in a colon cancer patient with a deficiency in MMR genes getting adjuvant chemotherapy.

EMH occurs when hematopoietic stem cells migrate out of the bone marrow to other areas of the body, typically as a result of bone marrow dysfunction or suppression. It is most often seen in hematological diseases but not frequently reported in the setting of solid tumor malignancies. The reasons for EMH in this subgroup of patients are not fully understood. Contributing factors include therapy-specific (growth factor support), bone marrow suppression secondary to chemotherapy and radiation therapy [4], and tumor-specific factors (cytokine and growth factors released by the tumor) [5]. Granulocyte colony-stimulating factor (G-CSF) promotes the proliferation and differentiation of neutrophils, as well as an increase in circulating hematopoietic progenitor cells leading to EMH [4]. In animal models, doxorubicin has been shown to cause EMH [6]. Histopathologically, MMR deficient tumors are characterized by having increased tumor-infiltrating lymphocytes and prominent inflammatory reactions [7, 8]. Studies have shown that MMR deficient tumors show signs of a high degree of infiltration with CD8+ cytotoxic T lymphocytes as well as helper T cells, leading to increased production of cytokines such as interferon-γ [9]. This could be a potential etiology behind EMH in our patient who was MMR deficient.

EMH in the setting of malignant solid tumors provides a diagnostic challenge that requires careful consideration and a high index of suspicion. EMH commonly involves the liver, spleen, and, occasionally, the lymph nodes. EMH can be microscopic or form masses [10] that appear in lymph nodes or in organs where metastasis is commonly discovered, which can potentially lead to incorrectly upstaging a patient’s malignancy. The imaging features of EMH are variable and difficult to discern from tumors, and, therefore, EMH can be easily misdiagnosed [2]. This highlights the necessity for pathologic confirmation by obtaining tissue. In most situations, the most likely explanation for a rising CEA, abnormal liver enzymes, and new hepatic lesions in a patient undergoing active treatment for colon cancer is the progression of colon cancer. But this should not be always assumed. The imaging findings of the hepatic lesions, in this case, were atypical of classic metastatic lesions and created enough uncertainty to lead to a biopsy of the lesions, which ultimately proved to be the vital piece of information needed to prevent unnecessary treatment. Furthermore, studies have shown that EMH does not affect the outcome of patients with solid organ malignancy and does not require EMH-specific treatment if asymptomatic [1, 2]. The outcome of our case also supports this recommendation as our patient also did not require any additional treatment for EMH; the liver lesions are decreasing in size based on the CT scans during the follow-up period.

## Conclusions

EMH is rare in colon cancer patients but requires a high degree of suspicion to avoid potentially toxic and unnecessary chemotherapy or other local therapy. Physicians should be aware of these phenomena and should keep a low threshold to perform a biopsy when in doubt. Patients who undergo chemotherapy for colon cancer typically do not require the use of recombinant growth factor support; moreover, the FOLFOX regimen has not been directly linked to causing EMH either, which makes a discovery in our case more unique. The role of the MMR-deficient state in the development of EMH is unclear and should be explored further.
